# Phenylacetic acid, an anti-vaginitis metabolite produced by the vaginal symbiotic bacterium *Chryseobacterium gleum*

**DOI:** 10.1038/s41598-024-62947-7

**Published:** 2024-05-28

**Authors:** Kang Mu Kwon, Eun-Hye Kim, Kyeong Hwa Sim, Youn Ju Lee, Eun-Ji Kang, Kap-Hoon Han, Jong-Sik Jin, Dae Keun Kim, Ji-Hye Ahn, In Hyun Hwang

**Affiliations:** 1 Department of Pharmacy, Woosuk University, Wanju, Jeonbuk 55338 Republic of Korea; 2 Department of Korean Pharmacy, Woosuk University, Wanju, Jeonbuk 55338 Republic of Korea; 3 Department of Pharmacology, School of Medicine, Daegu Catholic University, 33 Duryugongwon-ro 17-gil, Nam-gu, Daegu, 42472 Republic of Korea; 4 Department of Food and Biotechnology, Woosuk University, Wanju, Jeonbuk 55338 Republic of Korea; 5 Department of Pharmaceutical Engineering, Woosuk University, Wanju, Jeonbuk 55338 Republic of Korea; 6 Department of Oriental Medicine Resources, Jeonbuk National University, Iksan, Jeonbuk 54596 Republic of Korea; 7 Research Institute of Pharmaceutical Sciences, Woosuk University, Wanju, 55338 Republic of Korea

**Keywords:** Human microbiome, *Chryseobacterium gleum*, Phenylacetic acid, Vaginitis, Chemical ecology, Natural products, Small molecules

## Abstract

The human microbiome contains genetic information that regulates metabolic processes in response to host health and disease. While acidic vaginal pH is maintained in normal conditions, the pH level increases in infectious vaginitis. We propose that this change in the vaginal environment triggers the biosynthesis of anti-vaginitis metabolites. Gene expression levels of *Chryseobacterium gleum*, a vaginal symbiotic bacterium, were found to be affected by pH changes. The distinctive difference in the metabolic profiles between two *C. gleum* cultures incubated under acidic and neutral pH conditions was suggested to be an anti-vaginitis molecule, which was identified as phenylacetic acid (PAA) by spectroscopic data analysis. The antimicrobial activity of PAA was evaluated in vitro, showing greater toxicity toward *Gardnerella vaginalis* and *Candida albicans*, two major vaginal pathogens, relative to commensal *Lactobacillus* spp. The activation of myeloperoxidase, prostaglandin E_2_, and nuclear factor-κB, and the expression of cyclooxygenase-2 were reduced by an intravaginal administration of PAA in the vaginitis mouse model. In addition, PAA displayed the downregulation of mast cell activation. Therefore, PAA was suggested to be a messenger molecule that mediates interactions between the human microbiome and vaginal health.

## Introduction

Human health is closely associated with the microbiome of the human body (i.e., human microbiome)^1,2^. Small molecules produced by the human microbiome have been proposed to be involved in microbe–host and microbe–microbe interactions as key messengers^3^. The human urogenital microbiome harbors biosynthetic gene clusters that encode small molecules^4^. Lactocillin, an antibacterial compound active against vaginal pathogens, was isolated from the vaginal commensal bacterium *Lactobacillus gasseri*^4^. Changes in the composition of the vaginal microbiome were also observed in patients with vaginal infections, confirming the close relationship between vaginal health and the microbiome^5^. Probiotics for women’s urinary and vaginal health—various products that have been released to the market—are comprised of urogenital microbes, mainly including *Lactobacillus* spp.^6,7^. Despite the commercial success of these products, the molecular mechanisms underlying their biological activity remain to be identified, partly because the mass migration of microbes to the urogenital tract is unlikely when products are orally administered^6,7^.

The most common types of vaginitis are bacterial vaginosis, vulvovaginal candidiasis, and trichomoniasis, with *Gardnerella vaginalis*, *Candida albicans*, and *Trichomonas vaginalis* being the representative pathogens, respectively^5,8,9^. Because vaginitis symptoms are nonspecific, its diagnosis requires laboratory confirmation, which includes testing the pH of vaginal fluid^10^. A vaginal pH of greater than 4.5 is frequently observed in patients with infectious vaginitis, whereas healthy women maintain a pH level lower than 4.5^10,11^. Vaginal pH naturally increases during menopause due to hormonal changes and decreased lactobacilli composition, leading to the loss of natural epithelial defenses and, thereby, allowing the invasion of pathogens into the vagina and urinary tract^12,13^.

Clinical signs of vaginal inflammation, such as discharge, itching, and pain, are strongly associated with vaginitis^14^. In particular, vulvovaginal candidiasis is accompanied by pruritus, a prevalent symptom of allergic inflammation^14^. Mast cells are well-known for their crucial roles in itch sensation^15^. The stimulation of mast cells with various stimuli, including irritants and allergens, induces degranulation and the release of eicosanoids and pro-inflammatory cytokines^15^. Vaginal bacteria and/or their products were reported to activate mast cells to secrete mediators^16^.

Members of the genus *Chryseobacterium* are ubiquitous, but only a few are associated with colonization in humans, including *Chryseobacterium gleum* (formerly *Flavobacterium gleum*)^17–19^. *C. gleum* type strain F93 was originally isolated from a human high-vaginal swab^18,19^. The isolation of other *C. gleum* strains from patients^20^ has been reported as extremely rare cases, and their multidrug-resistant nature implies the potential pathogenicity of this species^21^. However, in addition to the low virulence of the genus *Chryseobacterium*^22^, the scarcity of clinical case reports and the weak biofilm-forming ability of *C. gleum*^23^ indicate that the presence of the species in clinical specimens is likely due to colonization rather than infection. Such unique viability of *C. gleum* led us to propose the hypothesis that its type strain responds to pathogenic infections by sensing a pH change in the human vaginal environment and produces defensive metabolites to provide competitive advantages.

Our previous study revealed that phenethylamine and *N*-acetylphenethylamine, discovered from the commensal oral microbe *Corynebacterium durum*, extended the lifespan of *Caenorhabditis elegans* by overexpressing SIR-2.1 protein^24^. In the present study, our continued efforts to search for chemical messengers from the human microbiome led to the identification of phenylacetic acid (PAA) from the vaginal symbiotic strain *C. gleum* F93. We also found that the production level of PAA varied considerably in response to pH changes. Furthermore, PAA displayed antimicrobial and anti-inflammatory activities in a mouse vaginitis model. Here, we describe our biochemical observations in detail and propose that PAA is a messenger molecule produced as a microbial defense mechanism against vaginitis.

## Results

### Isolation and identification of PAA

Based on the hypothesis that *C. gleum* produces messenger molecules for vaginal protection by sensing a pH increase, high-performance liquid chromatography (HPLC) data were compared between two acetone extracts of the microbe cultured at pH 5.5 and 7.3. Although a healthy human vaginal pH is 4.5 or less^10,11^, our small-scale pH screening results (data not shown) indicated that the lowest pH value for *C. gleum* was 5.5. This discrepancy could be attributed to the inherent differences between human vaginal fluid and the culture medium used in this study. Despite the variance in acidic pH levels, the impact of the shift between acidic and neutral pHs was evident in the experimental design, leading to changes in metabolite production.

**Figure 1:**
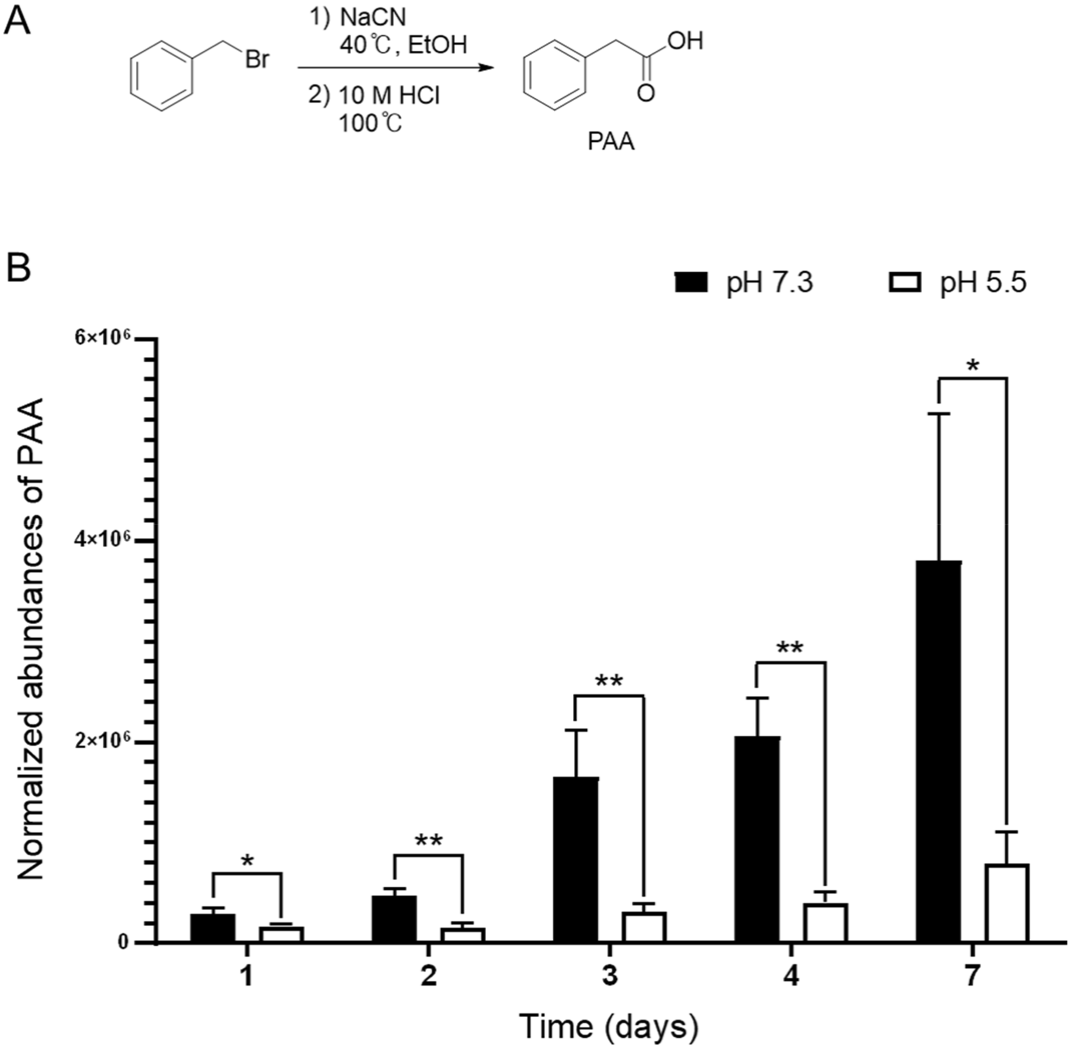
Chemical and biological synthesis of phenylacetic acid (PAA). (**A**) Synthetic scheme and chemical structure of PAA. (**B**) Relative abundance of PAA production by *C. gleum* cultured at pH 7.3 and 5.5. The MS peak areas of PAA were normalized by the corresponding OD_600_ values, and data were collected from three independent cultures at each time point and pH level (*P < 0.05, **P < 0.01).

Each extract was partitioned between water and organic solvent. PAA was observed predominantly in the water-soluble portion partitioned from pH 7.3 culture compared to pH 5.5, while the data obtained from the organic solvent-soluble portion of each extract were almost identical. Interpretation of the mass spectrometry (MS) and nuclear magnetic resonance (NMR) data enabled the identification of PAA, which was isolated by preparative HPLC. The molecular formula C_8_H_8_O_2_ was assigned to the compound based on HRESIMS and ^13^C NMR data. ^1^H NMR shift and integration values for multiplets at δ_H_ 7.24 and a singlet at δ_H_ 3.43 suggested the presence of mono-substituted benzene and an isolated methylene unit, respectively. The two partial structures were connected as a benzyl group by analysis of heteronuclear multiple-bond correlation (HMBC) data. In addition, an HMBC cross-peak of δ_H_ 3.43 to δ_C_ 173.4 indicated a linkage of the methylene group to a carboxyl carbonyl carbon, completing the structure of PAA, as shown in Fig. 1A. These NMR and MS data are consistent with literature reports of PAA^25–27^.

### Quantitative analysis of microbial PAA productions

The relative abundance of PAA generated by *C. gleum* cultured in acidic and neutral pHs was calculated to verify that pH was a crucial factor in PAA production. The culture supernatant was directly examined in triplicate at different time points by quantitative LC/MS analysis. The PAA peak areas were normalized by the optical density values of the corresponding cultures. More PAA was detected in the culture at pH 7.3 than at pH 5.5, and the gap between their PAA production levels widened with time until seven days of cultivation (Fig. 1B).

### RNA sequencing analysis

Based on the observation that external pH regulates metabolite production in *C. gleum*, its gene expression profiles at two different pH conditions were assessed by RNA-sequencing (RNA-seq) analysis. As a result, 1158 and 958 genes were differentially expressed by more than three-fold (log_2_) in pH 7.3 and 5.5 conditions, respectively, relative to the other pH condition (data not shown), indicating that pH is an important environmental regulator for *C. gleum*. The application of gene ontology (GO) analysis to the identified differentially expressed genes (DEGs) provided 856 annotated genes (Fig. S2, Supplementary Information 2). One of the most upregulated genes at pH 7.3, with high average counts per million reads (CPM), was RS19060, the gene product of which was annotated to phenylacetyl-CoA epoxidase subunit A. RS19070 and RS19080, encoding homologs of the phenylacetate-CoA oxygenase subunits PaaC and PaaJ, respectively, were observed at a nearby locus of RS19060 on the chromosome, suggesting that the genes related to PAA metabolism are clustered in *C. gleum*. Therefore, in conjunction with the chemical analysis data, the RNA-seq analysis results supported the effects of increasing pH on gene expression related to the biosynthesis and/or degradation of PAA. Although no studies on the biosynthetic pathways of PAA in *C. gleum* have been reported, these findings are consistent with the genes reportedly involved in PAA metabolism in other bacteria^28^. We also observed pH-dependent up- or down-regulation of genes related to pathways other than PAA synthesis, such as those involved in the anabolism of amino acids and nucleobases (Supplementary Information 3).

### Chemical synthesis of PAA

Because the natural product PAA was presumed to be a defensive messenger molecule produced by *C. gleum* by sensing pH increases, efforts toward the synthesis of PAA were made to examine its bioactivity against pathogens that cause vaginitis. The substitution reaction of benzyl bromide with sodium cyanide afforded benzyl cyanide (72%). Acidic hydrolysis of the nitrile group in benzyl cyanide with HCl enabled the production of phenylacetic acid (78%; overall 56%). The NMR and MS data of the synthetic product were identical to those observed for the natural product PAA^25–27^.

### Antimicrobial activity of PAA in vitro

**Figure 2:**
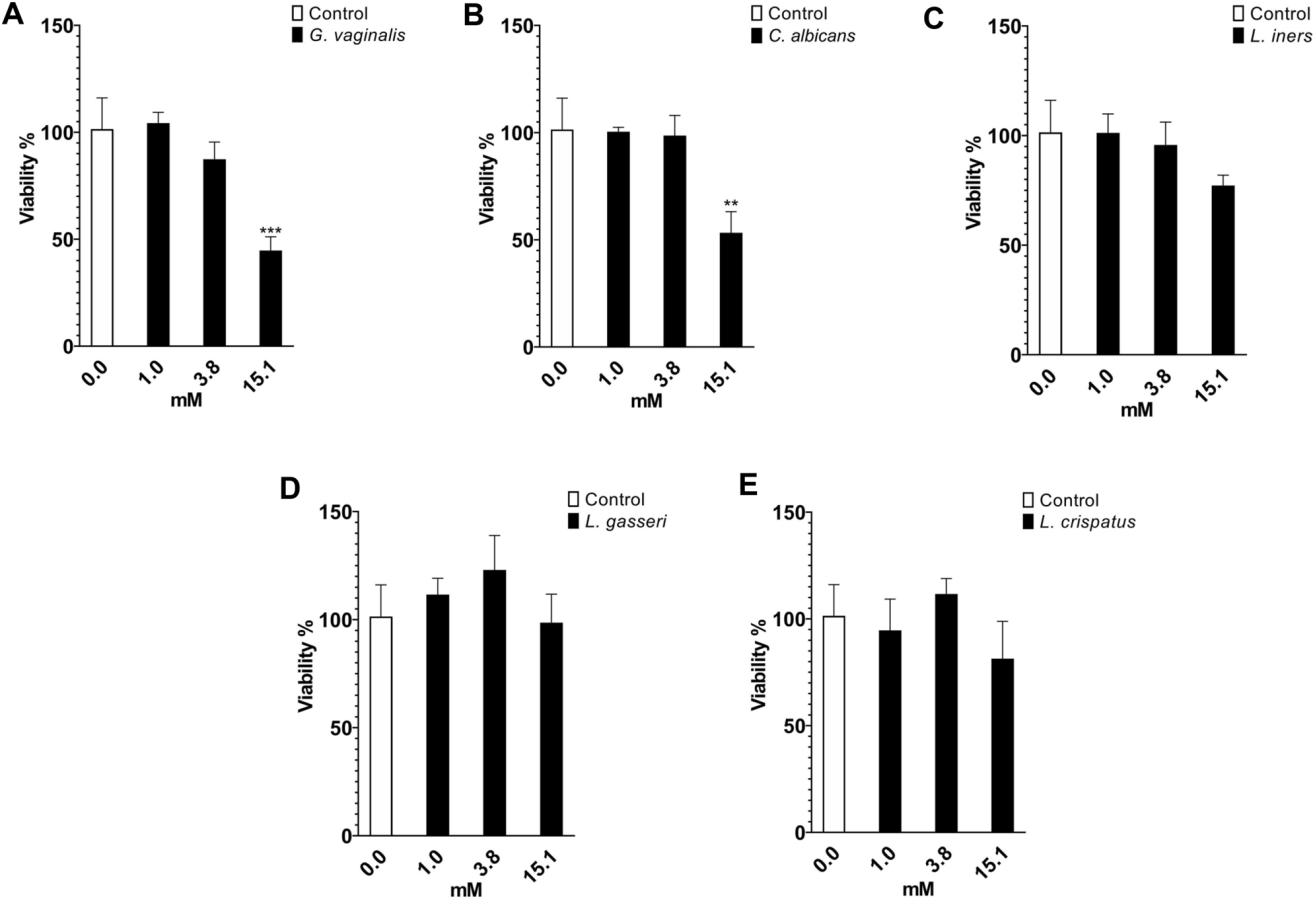
Relative viability of microorganisms upon treatment with PAA. The OD_600_ values of (**A**) *Gardnerella vaginalis*, (**B**) *Candida albicans*, (**C**) *Lactobacillus iners*, (**D**) *Lactobacillus gasseri*, and (**E**) *Lactobacillus crispatus* after treatment with PAA at concentrations of 1.0, 3.8, and 15.1 mM, converted to a percentage of the control. Data were collected from three independent cultures at each concentration (**P < 0.01, ***P < 0.001).

The observation of increased PAA production in the *C. gleum* culture incubated in neutral pH suggested that the compound was involved in a defensive mechanism under infected vaginal conditions. Among *Gardnerella* spp., *G. vaginalis* is often considered a representative pathogen associated with vaginitis, owing to well-documented virulence factors and frequent use in both in vitro and in vivo experiments for inducing bacterial vaginosis^29–31^. Additionally, *C. albicans* is a widely recognized major pathogen responsible for vulvovaginal candidiasis^10,32^. Therefore, optical density was measured upon treatment of *G. vaginalis* and *C. albicans* with PAA, showing inhibitory activity with IC_50_ values of 12.4 and 18.1 mM, respectively (Fig. 2A,B, Fig. S3). The activity of PAA against *Lactobacillus* spp. prevalent in the healthy vagina was also tested to evaluate the selectivity of the compound. The IC_50_ values of PAA toward *Lactobacillus iners*, *Lactobacillus gasseri*, and *Lactobacillus crispatus* were 26.3, 31.3, and 27.9 mM, respectively (Fig. 2C–E, Fig. S3).

### Effect of PAA on bacterial vaginosis and vulvovaginal candidiasis in mice

**Figure 3:**
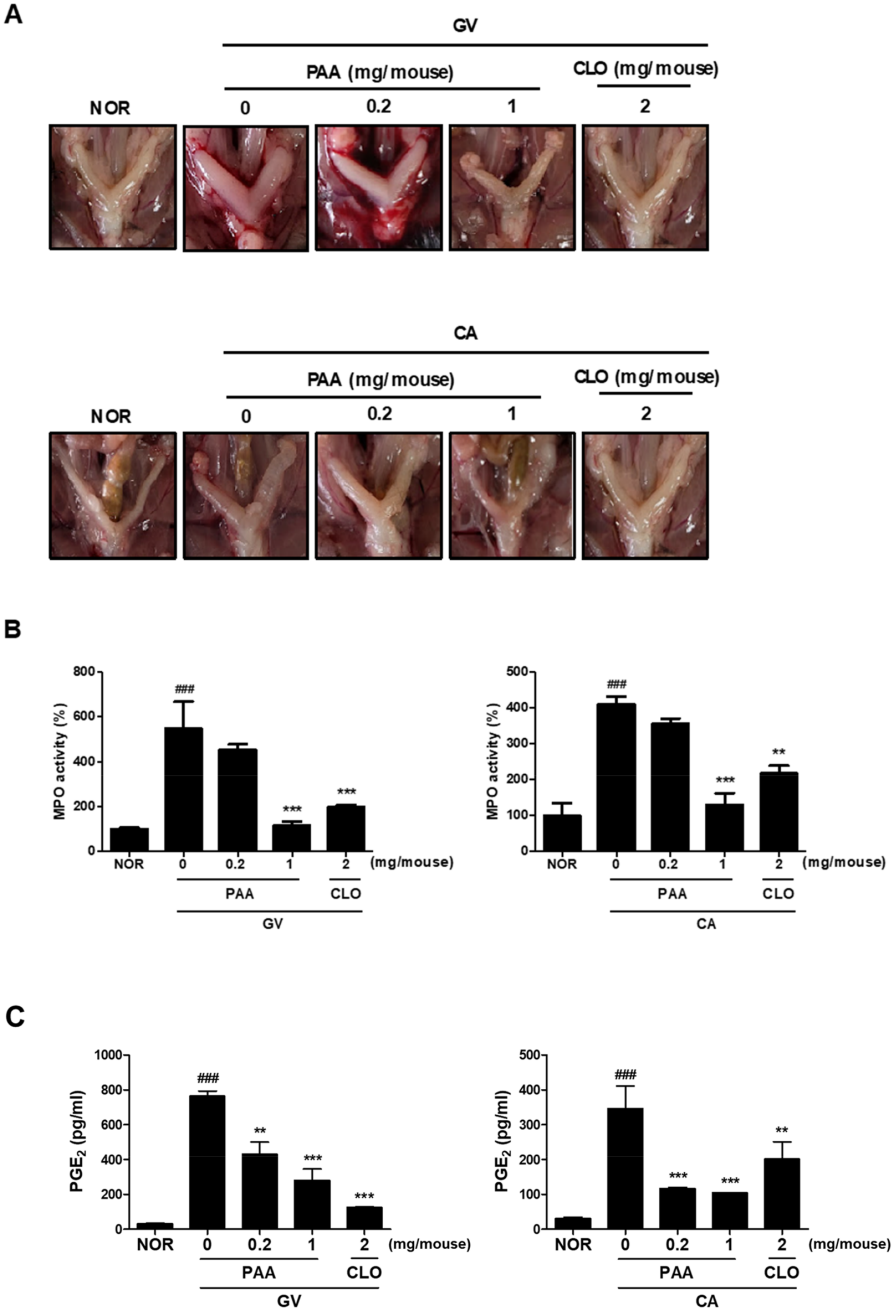

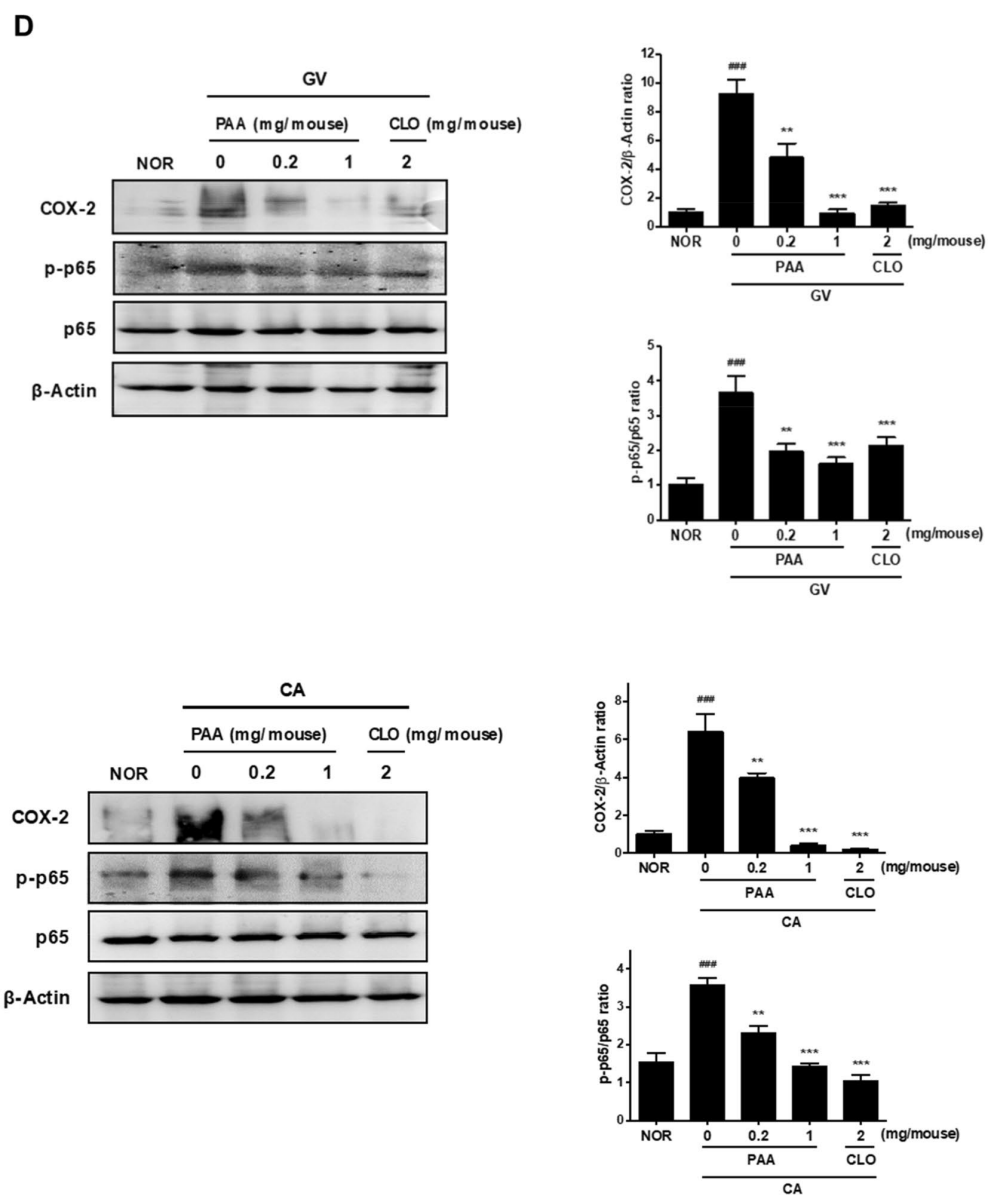
Effect of PAA on *Gardnerella vaginalis* (GV)- and/or *Candida albicans* (CA)-induced vaginitis and the expression of inflammatory markers in female mice. (**A**) Effect on GV- and/or CA-inflamed vagina and uterus. (**B**) Effect on myeloperoxidase (MPO) activity in vaginal tissues. (**C**) Production of PGE_2_. (**D**) Effect on COX-2 expression and NF-κB activation analyzed by Western blotting. β-Actin was used as an internal control. Female mouse vaginas were infected with GV and CA (both at 1 × 10^8^ CFU/mouse), except in the normal control group (NOR, normal group treated with vehicle alone). PAA and clotrimazole (CLO) were intravaginally administered once a day for 14 days. On day 15 post-infection, the mice were euthanized. The expression of COX-2 and activation of MPO, PGE_2_, and NF-kB were measured in vaginal tissues. Western blots from three independent results were quantified by densitometry using ImageJ. All values are shown as the mean ± SD of five replicate mice (n = 5). ^###^*P* < 0.001 *vs.* normal control group. **P < 0.01 and ***P < 0.001 *vs.* GV- and CA-treated control group.

Since *G. vaginalis* (GV) and *C. albicans* (CA) represent pathogens that cause bacterial vaginosis (BV) and vulvovaginal candidiasis (VVC), respectively^10,29–32^, we investigated the effects of PAA on GV- and CA-induced vaginitis in mice. Numerous studies have reported that myeloperoxidase (MPO) activity is used as a biomarker for GV- and CA-induced vaginitis, indicating an increased accumulation of polymorphonuclear cells in the vaginal tissue of mice^33^. In this regard, the vaginal inoculation of GV and CA in mice has been described to increase MPO activity and cause the development of BV and VVC, respectively^34–36^. A clinical study showed that clotrimazole was effective in the local treatment of mixed vaginal infections with BV and VVC^37^. Accordingly, we used clotrimazole as a positive control. As shown in Fig. 3A,B, GV and CA increased vulnerability to uterine horn infections, accompanied by the presence of uterine edema in these mice compared to the normal group. Interestingly, uterine inflammation and morphological changes induced by GV and CA were partially relieved by high-dose PAA treatment, although these changes did not fully return to normal levels. Moreover, GV- and CA-induced MPO activity was inhibited by the intravaginal administration of PAA at doses of 0.2 and 1 mg/mouse. In particular, PAA displayed greater inhibitory effects on MPO at 1 mg/mouse than clotrimazole at 2 mg/mouse in GV- and CA-treated mice, with levels close to those in the normal condition. Additionally, PAA significantly inhibited the GV- and CA-stimulated prostaglandin E_2_ (PGE_2_) levels in the vaginal tissue of mice (Fig. 3C). GV and CA vaginal infections also induced nuclear factor (NF)-κB p65 activation and cyclooxygenase (COX)-2 expression, both of which were significantly reduced by the PAA treatment (*p* < 0.05; Fig. 3D) in a dose-dependent manner. These results suggested that PAA attenuated the symptoms of BV and VVC through the NF-κB signaling pathway in mice.

### Effect of PAA on compound 48/80-stimulated mast cell activation

**Figure 4:**
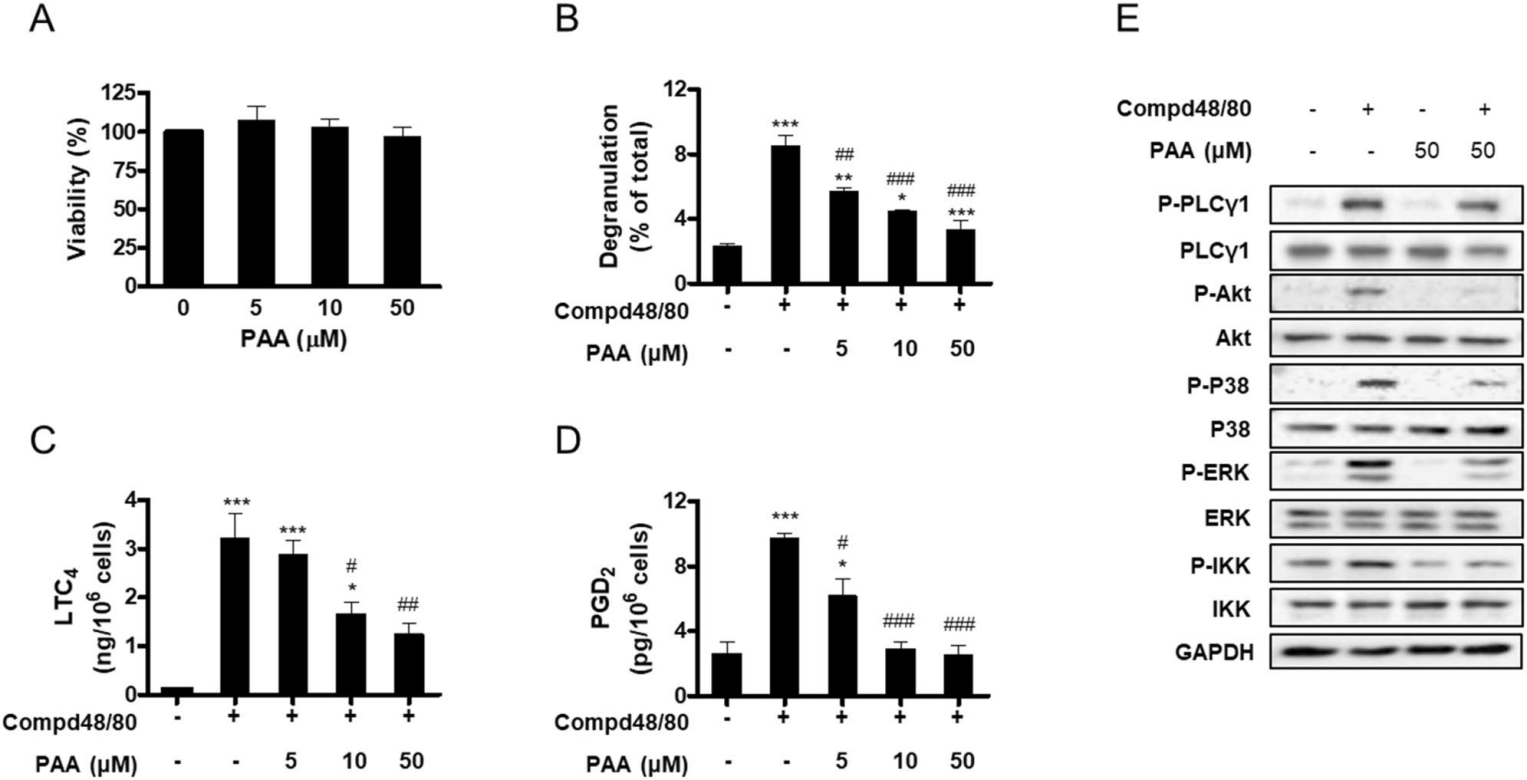
Effect of PAA on compound 48/80-stimulated mast cell activation. (**A**) HMC-1 cells were treated with PAA (0, 5, 10, and 50 μM) for 24 h. Cell viability was measured by the MTT assay. (**B–E**) HMC-1 cells were treated with different concentrations (0, 5, 10, and 50 μM) (**B–D**) or 50 μM (**E**) of PAA for 1 h, followed by stimulation with 30 μg/mL of compound 48/80 for 40 min. Degranulation (**B**) and secretion of LTC_4_ (**C**) and PGD_2_ (**D**) were measured. All values are shown as the mean ± SD from three independent experiments (*P < 0.05, **P < 0.01, and ***P < 0.001 *vs.* control; ^#^*P* < 0.05, ^##^*P* < 0.01, and ^###^*P* < 0.001 *vs*. compound 48/80 alone). Cell lysates were subjected to Western blotting with the indicated antibodies (**E**) and are representative of three independent experiments. GAPDH was used as an internal control.

We examined the effects of PAA on mast cell activation that leads to the allergic symptoms of vaginitis, such as itching and irritation. The treatment of HMC-1 cells with PAA concentrations up to 50 μM for 24 h had no effect on cell viability (Fig. 4A). HMC-1 cells were stimulated with compound 48/80 in the presence or absence of different concentrations of PAA (0, 5, 10, and 50 μM). Then, degranulation and eicosanoid production levels were measured by the β-hexosaminidase assay and immunoassay, respectively. PAA decreased compound 48/80-stimulated degranulation (Fig. 4B) and leukotriene C_4_ (LTC_4_) and prostaglandin D_2_ (PGD_2_) production (Fig. 4C,D) in a concentration-dependent manner. Consistent with the inhibitory effect of PAA on mast cell activation, 50 μM PAA reduced the activation of PLCγ1, Akt, p38, ERK1/2, and IKK, which are positive signaling molecules involved in mast cell activation (Fig. 4E). Therefore, PAA was suggested to act as a down-regulator of mast cell activation.

## Discussion

The dysbiosis of vaginal microbiota is closely related to vaginitis^38^, most cases of which are induced by an overgrowth of *G. vaginalis* and *C. albicans*^10,29–32^. BV and VVC require oral or local treatment with antibiotics and antifungals, respectively, based on pathological findings. However, conventional treatment for genital infections has faced many challenges, which include increasing microbial resistance, adverse effects, and recurrent infections^39^. Recently, vaginal microbiota transplantation (VMT) in patients with recurrent BV showed the long-term recovery of *Lactobacillus* spp. dominance, as in healthy vaginas^40^. Therefore, the human microbiome has become an emerging source of new antimicrobial agents that maintain balanced microbial communities.

One diagnostic criterion for vaginitis is the pH of vaginal fluid. Vaginal pH greater than 4.5 is frequently observed in patients with BV, acute vaginal *Candida* infection, and trichomoniasis, whereas healthy women maintain a pH level lower than 4.5^10,11^. In addition to the laboratory-based pH test, a microbiological approach has been recently recommended for the comprehensive diagnosis of vaginitis^41^. Furthermore, the presence of two different types of vaginitis, called mixed vaginitis, causes vaginal symptoms and affects the health of women of all ages worldwide^37,42^. Among the various types of mixed vaginitis, the combination of BV and VVC is the most prevalent clinical presentation^37^. Based on the reports that the human microbiota provides chemical messengers for microbe-host and microbe-microbe interactions^4,5^, we postulated that defensive compounds are produced by the vaginal symbiotic bacterium *C. gleum* in response to a pathogenic invasion by sensing the pH change.

In the present study, *C. gleum* collected from a human high-vaginal swab was incubated at pH 5.5 and 7.3 to simulate healthy and vaginitis conditions, respectively. Comparison of the HPLC profiles of the two different cultures revealed distinctive differences, which were attributed to PAA by NMR and MS interpretation. The quantitative analysis of PAA produced by *C. gleum* indicated that its abundance was significantly higher in pH 7.3 conditions over the course of a week relative to pH 5.5. Although the genomic sequencing of *C. gleum* has not been completed, its draft genome available in public databases enabled the analysis of RNA-seq data. Different pH conditions changed various gene expressions, including the genes involved in the biosynthesis and/or degradation of PAA, leading to the accumulation of PAA in pH 7.3 conditions. Also, we found that the genes related to PAA metabolism in *C. gleum* might be clustered in the genome, suggesting the coregulation of their expression by the signal perception of environmental cues, such as pH changes. The chemical synthesis of PAA was achieved in two steps, allowing the examination of the biological activity of the compound against microbes related to vaginitis. PAA showed antimicrobial activity against the two representative vaginal pathogens, *G. vaginalis* and *C. albicans,* with IC_50_ values of 12.4 and 18.1 mM, respectively, with weaker effects on *L. iners*, *L. gasseri*, and *L. crispatus*. Interestingly, although most vaginal *Lactobacillus* spp. are known to be beneficial, the contribution of *L. iners* to vaginal health has been debated^43^. Our in vitro study showed that the reduced viability of *L. iners* was not statistically significant.

In addition to the reported antimicrobial activity^26,27^, PAA has been described to play a messenger role in various ecosystems. The bacterium *Phaeobacter gallaeciensis* produces PAA to mediate bacteria-algae interactions, in which PAA converts to roseobacticides, antibiotic compounds that protect an environmentally valuable marine microalga^44^. PAA was also proposed to be involved in the interaction between fungal pathogens and rhizobacterial communities. The invasion of plant roots with the fungus *Rhizoctonia solani* induced a shift in the bacterial composition of the rhizosphere and secondary metabolite production, including PAA, as a part of the defensive mechanisms^45^.

Vaginitis symptoms, including vaginal discharge, itching, and burning, are closely associated with inflammatory responses^46^. Diclofenac is a nonsteroidal anti-inflammatory drug (NSAID) that contains a PAA moiety as a pharmacophore^47^. The mechanism of action of NSAIDs involves the inhibition of COX, an enzyme that oxidizes arachidonic acid to prostanoids. Currently, a new formulation that combines clotrimazole with diclofenac (ProF-001) is under phase 3 clinical trials for recurrent VVC^48^. In addition, the topical application of ibuprofen, another representative NSAID with a PAA structure, was reported to reduce the symptoms of vaginitis in clinics^49^. A recent study revealed that patients with chronic idiopathic vaginitis responded well to mast cell-targeted treatment^50^, indicating that mast cells are involved in the pathophysiology of vaginitis. In the present study, PAA was able to reduce compound 48/80-stimulated degranulation and LTC_4_ and PGD_2_ production in mast cells, as well as the production of PGE_2_ and expression of COX-2 in mouse vaginal tissues infected with BV and VVC. Therefore, the anti-vaginitis activity of PAA was suggested to be mediated by the downregulation of mast cell activation and PGE_2_/COX-2 expression.

In summary, the vaginal symbiotic bacterium *C. gleum* was found to produce PAA, which was significantly enhanced by a pH increase similar to the physiological condition of vaginal infections. Notably, we demonstrated that the intravaginal administration of PAA was effective in reducing GV- and CA-induced vaginal symptoms and MPO activity in vivo. PAA was also able to inhibit GV- and CA-induced NF-κB and PGE_2_ activation and COX-2 expression, indicating that its anti-vaginitis effect was partly due to the modulation of immune responses in the vagina. Moreover, PAA attenuated compound 48/80-stimulated mast cell activation determined by degranulation and eicosanoid production. Our findings suggest the potential role of PAA in treating BV and VVC, as well as chronic vaginitis caused by abnormal mast cell activation^50^. These results support our hypothesis that PAA production might be involved in the mechanism that protects the vaginal microenvironment from pathogenic invasion as a part of microbe–host interactions. However, its mechanistic actions remain to be elucidated.

## Materials and methods

### General experimental procedures

Optical density was recorded at 600 nm using an ELISA microplate reader (Tecan Sunrise, Grödig, Austria). A Gilson system (Gilson Medical Electronics, Middleton, WI, USA), equipped with Gilson 305 and 306 pumps and a Gilson 151 UV/Vis detector, was connected to either a Gemini C_18_ (Phenomenex, 5 μm, 10.0 × 250 mm) or Luna C_18_ (Phenomenex, 15 μm, 21.2 × 250 mm) column for HPLC separation. Flash column chromatography was carried out using a CombiFlash Retrieve system (Teledyne Isco, Lincoln, NE, USA) with a RediSep silica gel prepacked (12 g, 20 × 80 mm) column. LC/MS data measurements were conducted on an AQUITY Arc UHPLC system (Waters, Milford, MA, USA) connected to a ZQ single quadrupole detector with an XBridge BEH C_18_ (Waters, 2.5 µm, 2.1 × 150 mm) column. NMR spectra were obtained on JNM-ECZ500R and -ECZ600R spectrometers (JEOL, Tokyo, Japan). The strict anaerobes were cultured in a VS-5600A anaerobic chamber (Vision Bionex, Bucheon, Republic of Korea). The culture media, brain heart infusion (BHI) and tryptic soy broth, were purchased from BD Biosciences (San Jose, CA, USA), and Penassay and MRS broths were acquired from Sigma-Aldrich (St. Louis, MO, USA). Compound 48/80 was obtained from Sigma-Aldrich.

### Microbial sources

*C. gleum* type strain F93, isolated from a human high-vaginal swab collected in London^18,19^, was provided by the Korean Collection for Type Cultures (KCTC) under the accession number KCTC 2904. The microbial strains used for antimicrobial activity were *G. vaginalis* (KCTC 5097), *C. albicans* (KCTC 7270), *L. iners* (KCTC 15516), *L. gasseri* (KCTC 3163), and *L. crispatus* (KCTC 3178).

### Extraction

The vaginal bacterial strain *C. gleum* F93 was cultured in two groups, both in BHI medium for four weeks at 37 °C. The pH of one group (4 × 1 L) was adjusted to 5.5 by adding HCl, while the other group (4 × 1 L) was incubated without HCl (pH 7.3). Adsorption of the organic materials in the culture was accomplished by adding sterilized XAD-7-HP resin (20 g/L), followed by shaking the mixture at 200 rpm for 2 h. The resin collected by filtration through cheesecloth was extracted with acetone to afford 11.0 g (pH 5.5) and 21.1 g (pH 7.3) of brown residue.

### Isolation of PAA

The two acetone extracts of *C. gleum* cultured at pH 5.5 (11.0 g) and pH 7.3 (21.1 g) were partitioned between CHCl_3_ (3 × 40 mL; 599 mg for pH 5.5; 454 mg for pH 7.3) and H_2_O (40 mL; 5.8 g for pH 5.5; 10.2 g for pH 7.3). The removal of lipids from the resulting set of organic phase-soluble portions was accomplished by partitioning between *n*-hexane (3 × 16 mL; 64.8 mg for pH 5.5; 37.8 mg for pH 7.3) and MeCN (16 mL; 186 mg for pH 5.5; 190 mg for pH 7.3). A set of the materials soluble in the same solvent was compared using C_18_ HPLC (5 μm, 10.0 × 250 mm, 2 mL/min) with MeCN/H_2_O containing 0.1% formic acid as a mobile phase. PAA (*t*_R_ 28.5 min; 2.5 mg; overall yield 0.94%) was purified from the H_2_O-soluble material (129 mg out of 10.2 g) of *C. gleum* extract cultured at pH 7.3 by C_18_ HPLC (15 μm, 21.2 × 250 mm, 8 mL/min), eluting with 10% MeCN/H_2_O with 0.1% formic acid for 10 min, followed by a linear gradient to 100% over 20 min.

### Synthesis of PAA

Benzyl bromide (500 mg, 1 equiv.) and NaCN (287 mg, 2 equiv.) were dissolved in 95% EtOH (10 mL), and the reaction was stirred overnight at 40 °C. The resulting mixture was partitioned between EtOAc (15 mL) and H_2_O (3 × 15 mL). The organic phase was collected, and the solvent was removed by vacuum evaporation to afford benzyl cyanide (246 mg, 72%). After adding 10 M HCl (7 mL) to benzyl cyanide (246 mg), the solution was stirred and refluxed overnight at 100 °C. The resulting product was extracted with CHCl_3_ (3 × 15 mL), and the organic solvent was evaporated to provide 223 mg (78%; overall 56%) of PAA. Samples of PAA for biological tests were purified by flash column chromatography on silica gel (20 × 80 mm) and eluted with *n*-hexane/ethyl acetate (4:1).

### LC–MS analysis of PAA production levels

BHI liquid media at pH 5.5 and pH 7.3 was produced with and without HCl, respectively. After autoclave sterilization, 198 mL of each medium was inoculated with 2 mL of *C. gleum* culture (OD_600_ = 0.1 ± 0.02). The resulting cultures were shaken at 120 rpm at 37 °C. At each time point, OD_600_, pH, and LC–MS data of the samples collected from each *C. gleum* culture were recorded. The relative abundance of PAA (*t*_R_ = 6.23 min) was calculated by LC–MS (2.5 µm, 2.1 × 150 mm, 0.4 mL/min), eluted with 15% MeCN/H_2_O (0.1% formic acid) for 1.5 min, followed by a linear gradient to 100% MeCN/H_2_O (0.1% formic acid) over 8.5 min. The electrospray ionization mass spectrometry (ESIMS) was set to the negative ion mode with a capillary voltage of 3.00 kV, cone voltage of 60 V, desolvation gas flow of 500 L/h, cone gas flow of 50 L/h, desolvation temperature of 400 °C, and source temperature of 120 °C.

### RNA sequencing

Total RNA concentration was calculated by Quant-IT RiboGreen (Invitrogen, #R11490). Samples were run on TapeStation RNA ScreenTape (Agilent) to assess the integrity of the total RNA. Only high-quality RNA preparations, with an RNA integrity number (RIN) greater than 7.0, were used for RNA library construction. The libraries were independently prepared with 1 μg of total RNA for each sample using the Illumina TruSeq Stranded mRNA Sample Prep Kit (Illumina, Inc., San Diego, CA, USA, #RS-122-2101). The libraries were quantified using KAPA Library Quantification Kits for Illumina Sequencing platforms according to the qPCR Quantification Protocol Guide (KAPA), and qualified using TapeStation D1000 ScreenTape. Indexed libraries were then submitted to Illumina HiSeqXten, and paired-end (2 × 151 bp) sequencing was performed by Macrogen Incorporated (Seoul, Republic of Korea).

### Statistical analysis of gene expression levels

The gene model of the *C. gleum* genome was obtained from the National Center for Biotechnology Information (NCBI) public database. The RNA-seq results matched with the *C. gleum* genomic data were analyzed using InfoBoss and Macrogen pipelines for obtaining gene expression levels. Briefly, the relative abundance of genes was measured in read counts using HTSeq. Statistical analysis was performed to identify DEGs using the abundance estimates for each gene in the samples. The statistical significance of the differential expression data was determined by nbinomWaldTest using DESeq2 and fold change, in which the null hypothesis was that no difference exists among groups. For the DEG set, hierarchical clustering analysis was performed using complete linkage and Euclidean distance as a measure of similarity. Gene enrichment and functional annotation analysis, and pathway analysis for the significant gene list were performed based on blastGO (http://geneontology.org/) and the Kyoto Encyclopedia of Genes and Genomes (KEGG; http://kegg.jp).

### Antimicrobial activity in vitro

All microbes used for antimicrobial activity were incubated at 37 °C in tryptic soy, Penassay, and MRS broth for *C. albicans*, *G. vaginalis*, and *Lactobacillus* spp., respectively. Anaerobic and 5% CO_2_ conditions were applied for *Lactobacillus* spp. and *G. vaginalis* cultures, respectively. A stock solution of PAA was prepared in 20% polyethylene glycol (PEG) in sterile water. Serial dilutions of the PAA stock solution were made in a liquid medium, and 50 μL was added to each well of 96-well plates. The microbial culture was adjusted to an OD_600_ of 0.1 ± 0.02, and 50 μL was transferred to each well containing PAA solution, producing final concentrations of PAA ranging from 0.2 to 30.3 mM (in 0.02% to 2.5% PEG). The plate was sealed with cover film and incubated at 37 °C for 20 h prior to measuring OD_600_ values.

### Murine vaginal infection models

Female C57BL/6 mice (8 weeks old) were purchased from Orient Bio Inc. (Seongnam, Republic of Korea). All mice were housed in a room with standard environmental conditions (12/12 h light–dark cycle, temperature 22 ± 2 °C, and humidity 50–60%) and provided food and water ad libitum. To overcome the limitation of mouse models and closely mimic clinical situations in humans, the mice were pretreated with β-estradiol-3-benzoate before infection to render them immunocompromised. β-Estradiol-3-benzoate (0.125 mg/mouse) was intraperitoneally injected into all mice three days before inoculation with *G. vaginalis* or *C. albicans*. The mice were intravaginally injected with *G. vaginalis* or *C. albicans* (both at 1 × 10^8^ CFU/20 µL phosphate-buffered saline, PBS), except for the normal control group. In our preliminary experiment, the administration of PAA at 1 mg/mouse did not induce toxicity or inflammation in the vaginal tissue of mice (Fig. S9). Based on these findings, we opted to use the low and high concentrations, namely 0.2 and 1 mg/mouse, for further investigation. PAA (0.2 and 1 mg/mouse) and clotrimazole (2 mg/mouse) were intravaginally administered once a day for 14 days beginning the day after infection. The vaginas were washed with PBS, and the washed and excised vaginas were stored at − 80 °C for MPO activity and immunoblotting analyses. Euthanasia was performed using cervical dislocation. Animal treatment and maintenance for this study were approved by the Institutional Animal Care and Use Committee (IACUC) of Woosuk University (protocol number WS-2023-11). All methods were performed in accordance with the relevant guidelines and regulations. The study is reported in accordance with ARRIVE guidelines (https://arriveguidelines.org).

### ELISA

To analyze MPO activity and PGE_2_ production, commercial ELISA kits were used. Vaginal tissue lysate was prepared by homogenizing the tissue in RIPA lysis buffer (Biosesang, Seoul, Republic of Korea) containing phosphatase and protease inhibitors. The supernatant was added to a reaction mixture containing 1.6 mM tetramethyl benzidine and 0.1 mM hydrogen peroxide, incubated at 37 °C, and the absorbance at 650 nm was measured over time. The MPO activity assay and PGE_2_ ELISA were performed according to the manufacturer’s instructions.

### Western blot analysis

Vaginal tissues were homogenized in RIPA buffer to extract protein. Protein concentrations were measured by the Bradford assay. The protein samples were mixed with 5× sodium dodecyl sulfate (SDS) sample buffer, boiled for 5 min, and separated by 10% SDS–polyacrylamide gel electrophoresis. After electrophoresis, the proteins were transferred to membranes. The membranes were blocked with 5% skimmed milk for 30 min. After washing with Tris-buffered saline (TBS) containing Tween-20 (TBS-T, 0.1%), the membranes were incubated overnight at 4 ℃ with specific primary antibodies in 5% skimmed milk. The membranes were washed three times with TBS-T before and after incubation with secondary antibodies (1:1000–2500) for 2 h at room temperature. The immunopositive bands were visualized by enhanced chemiluminescence and exposed to Image Quant LAS-4000.

### Cell culture and activation of mast cells

Human mast cells (HMC-1) were cultured in Iscove’s Modified Dulbecco’s Medium (Gibco) supplemented with 10% fetal bovine serum and 1% penicillin/streptomycin. For cell stimulation, HMC-1 cells were treated with 30 μg/mL of compound 48/80 for 40 min.

### β-Hexosaminidase assay

The level of degranulation was measured by the β-hexosaminidase assay. The culture medium was collected, and 25 μL of supernatant was incubated with 50 μL of *p*-nitrophenyl-*N*-acetyl-β-d-glucosaminide (1.3 mg/mL) at 37 °C for 1 h. The enzymatic reaction was stopped by adding 0.2 M glycine buffer (pH 10.7). The absorbance was measured at 405 nm on a SpectraMax iD5 microplate reader (Molecular Devices, San Jose, CA, USA). Data are expressed as a percentage of the total values.

### Measurement of eicosanoid production

The production of PGD_2_ and LTC_4_ was measured as described previously^51^. The culture medium was collected, and the levels of PGD_2_ and LTC_4_ were quantified using the respective eicosanoid immunoassay kits (Cayman Chemicals, Ann Arbor, MI, USA) according to the manufacturer’s instructions.

### MTT assay

Cell viability was measured by the MTT assay. Cells seeded on 96-well plates (1 × 10^6^ cells/100 μL/well) were treated with PAA for 24 h. After adding tetrazolium salt 3-[4,5-dimethylthiazol-2-yl]-2,5-diphenyltetrazolium bromide (0.5 mg/mL), the plates were incubated at 37 °C for 3 h. Then, 100 μL of dimethyl sulfoxide was added to each well, and the absorbance was measured at 595 nm.

### Statistical analysis

The data are presented as the mean ± SD. Normality and homogeneity of variances were assessed using the Shapiro–Wilk test and Levene’s test, respectively. An independent *t*-test was conducted when the assumptions of sample independence, normality, and homogeneity of variance were satisfied; otherwise, statistical significance was evaluated using the Kruskal–Wallis H test followed by Dunn–Bonferroni post hoc analysis. All statistical analyses were performed using R statistical software (version 4.3.1; R Foundation for Statistical Computing, Vienna, Austria). Significance was defined as *P < 0.05, **P < 0.01, ***P < 0.001. Data visualization was carried out using GraphPad Prism software (version 8.2).

### Supplementary Information

Supplementary Information 1.

Supplementary Information 2.

Supplementary Information 3.

Supplementary Information 4.

## Data Availability

The raw RNA sequencing data are available in Sequence Read Archive (SRA) database with accession numbers SRR25567728 and SRR25567729 (BioProject PRJNA1003387).
