# Supplementary material for: Phenylacetic acid, an anti-vaginitis metabolite produced by the vaginal symbiotic bacterium Chryseobacterium gleum
Source: Sci Rep. 2024 May 28;14:12226. doi: 10.1038/s41598-024-62947-7 (PMC11133378; doi:10.1038/s41598-024-62947-7)
Supplement: Supplementary file 1 — Supplementary Information 1. [file 41598_2024_62947_MOESM1_ESM.docx]

**Supplementary information**

**Phenylacetic acid: An anti-vaginitis metabolite produced by the vaginal symbiotic bacterium *Chryseobacterium gleum***

Kang Mu Kwon^1^, Eun Hye Kim^2^, Kyeong Hwa Sim^3^, Youn Ju Lee^3^, Eun Ji Kang^4^, Kap-Hoon Han^5^, Jong-Sik Jin^6^, Dae Keun Kim^1,7^, Ji-Hye Ahn*^,2^, and In Hyun Hwang*^,1,7^

^1^Department of Pharmacy, Woosuk University, Wanju, Jeonbuk 55338, Republic of Korea

^2^Department of Oriental Pharmacy, Woosuk University, Wanju, Jeonbuk 55338, Republic of Korea

^3^Department of Pharmacology, School of Medicine, Daegu Catholic University, 33 Duryugongwon-ro 17-gil, Nam-gu, Daegu 42472, Republic of Korea

^4^Department of Food and Biotechnology, Woosuk University, Wanju, Jeonbuk 55338, Republic of Korea

^5^Department of Pharmaceutical Engineering, Woosuk University, Wanju, Jeonbuk 55338, Republic of Korea

^6^Department of Oriental Medicine Resources, Jeonbuk National University, Iksan, Jeonbuk 54596, Republic of Korea

^7^Research Institute of Pharmaceutical Sciences, Woosuk University, Wanju 55338, Republic of Korea

*Address correspondence to Ji-Hye Ahn, jihyeahn20@woosuk.ac.kr; In Hyun Hwang, inhyun.hwang@woosuk.ac.kr

**Supplementary information**

| No. | Contents | Page |
| --- | --- | --- |
| Table S1 | Normalized abundance of PAA produced by *C. gleum* cultured at pH 7.3 and 5.5 | 4 |
| Table S2 | Relative viability of vaginal microbes upon treatment with PAA | 5 |
| Figure S1 | MS peak areas of PAA (A) and OD600 values (B) over time of *C. gleum* cultures at pH 7.3 and 5.5 | 6 |
| Figure S2 | Differentially expressed genes (DEGs) of *C. gleum* after cultivation at pH 5.5 or 7.3 | 7 |
| Figure S3 | Survival curves of vaginal pathogens and commensal *Lactobacillus* spp. upon treatment with PAA | 8 |
| Figure S4 | HRESIMS data of PAA | 9 |
| Figure S5 | ^1^H NMR spectrum of PAA (500 MHz, DMSO-*d*_6_) | 10 |
| Figure S6 | ^13^C NMR spectrum of PAA (125 MHz, DMSO-*d*_6_) | 11 |
| Figure S7 | HMQC spectrum of PAA (500 MHz, DMSO-*d*_6_) | 12 |
| Figure S8 | HMBC spectrum of PAA (500 MHz, DMSO-*d*_6_) | 13 |
| Figure S9 | Effects of PAA on vaginal infection and expression of inflammatory markers in female mice | 14 |

**Table S1. Normalized abundance*^a^* of PAA produced by *C. gleum* cultured at pH 7.3 and 5.5**

| **pH** | **No.** |  | **Time (days)** | | | | |
| --- | --- | --- | --- | --- | --- | --- | --- |
|  |  |  | **1** | **2** | **3** | **4** | **7** |
| **7.3** | **A** | MS peak area | 114,093 | 175,058 | 775,346 | 970,039 | 2,354,734 |
|  |  | OD_600_ | 0.344 | 0.433 | 0.438 | 0.452 | 0.595 |
|  |  | Normalized abundance | **331,666** | **404,758** | **1,769,187** | **2,144,681** | **3,960,865** |
|  | **B** | MS peak area | 169,233 | 294,964 | 1,109,648 | 1,554,011 | 3,298,483 |
|  |  | OD_600_ | 0.519 | 0.566 | 0.541 | 0.650 | 0.638 |
|  |  | Normalized abundance | **325,887** | **521,138** | **2,050,158** | **2,390,786** | **5,170,036** |
|  | **C** | MS peak area | 57,914 | 201,353 | 482,762 | 720,039 | 1,214,239 |
|  |  | OD_600_ | 0.249 | 0.398 | 0.426 | 0.438 | 0.531 |
|  |  | Normalized abundance | **232,586** | **506,548** | **1,134,576** | **1,645,803** | **2,286,702** |
|  |  | **Mean normalized abundance ± SD** | **296,713 ± 55,610** | **477,482 ± 63,401** | **1,651,307 ± 469,036** | **2,060,423 ± 379,571** | **3,805,868 ± 1,447,902** |
| **5.5** | **D** | MS peak area | 42,073 | 67,870 | 133,114 | 178,223 | 583,966 |
|  |  | OD_600_ | 0.221 | 0.325 | 0.343 | 0.341 | 0.518 |
|  |  | Normalized abundance | **190,117** | **208,959** | **388,654** | **522,189** | **1,127,347** |
|  | **E** | MS peak area | 42,788 | 38,784 | 89,729 | 129,479 | 348,457 |
|  |  | OD_600_ | 0.276 | 0.366 | 0.379 | 0.389 | 0.703 |
|  |  | Normalized abundance | **155,141** | **105,967** | **236,908** | **333,022** | **495,812** |
|  | **F** | MS peak area | 24,400.0 | 48,397.0 | 103,064.0 | 120,912.0 | 252,694.0 |
|  |  | OD_600_ | 0.146 | 0.336 | 0.325 | 0.333 | 0.337 |
|  |  | Normalized abundance | **167,698** | **144,124** | **317,609** | **363,099** | **750,948** |
|  |  | **Mean normalized abundance ± SD** | **170,986 ± 17,718** | **153,017 ± 52,069** | **314,390 ± 75,924** | **406,103 ± 101,652** | **791,369 ± 317,702** |

*^a^*The MS peak area of PAA in each *C. gleum* culture was normalized by the OD_600_ value of the corresponding culture.

**Table S2. Relative viability*^a^* of vaginal microbes upon treatment with PAA**

|  | PAA conc. (mM) | | | | | | | | | IC50 (mM) |
| --- | --- | --- | --- | --- | --- | --- | --- | --- | --- | --- |
|  | 0.0 | 0.2 | 0.4 | 1.0 | 1.9 | 3.8 | 7.6 | 15.1 | 30.3 |  |
| *G. vaginalis* | 100.0  ± 0.0 | 106.5  ± 8.0 | 104.5  ± 8.9 | 104.3  ± 5.0 | 92.8  ± 10.2 | 87.4  ± 8.0 | 64.5  ± 13.2 | 44.7  ± 6.4 | 46.2  ± 5.6 | 12.4 |
| *C. albicans* | 100.0  ± 0.0 | 100.6  ± 9.6 | 100.0  ± 8.2 | 100.4  ± 2.1 | 92.4  ± 6.9 | 98.6  ± 9.4 | 94.3  ± 11.0 | 53.3  ± 9.9 | 28.6  ± 1.9 | 18.1 |
| *L. iners* | 100.0  ± 0.0 | 99.0  ± 6.0 | 97.8  ± 4.9 | 101.2  ± 8.6 | 95.3  ± 13.3 | 95.7  ± 10.4 | 92.8  ± 10.1 | 77.2  ± 4.7 | 42.0  ± 6.1 | 26.3 |
| *L. gasseri* | 100.0  ± 0.0 | 101.7  ± 3.0 | 108.4  ± 6.2 | 111.6  ± 7.5 | 108.7  ± 6.4 | 123.0  ± 15.9 | 118.6  ± 17.4 | 98.6  ± 13.2 | 55.2  ± 5.1 | 31.3 |
| *L. crispatus* | 100.0  ± 0.0 | 100.8  ± 12.6 | 97.6  ± 10.5 | 94.6  ± 14.7 | 96.3  ± 14.9 | 111.7  ± 7.2 | 102.6  ± 13.1 | 81.4  ± 17.4 | 45.3  ± 6.2 | 27.9 |
| PEG (vehicle) | 100.0  ± 0.0 | 101.4  ± 8.4 | 102.6  ± 8.6 | 104.3  ± 10.1 | 101.5  ± 13.0 | 105.3  ± 12.6 | 102.2  ± 13.5 | 94.8  ± 18.2 | 90.9  ± 20.0 |  |

*^a^*OD_600_ values were converted to a percentage of the control. Data were collected from three independent cultures at each concentration.


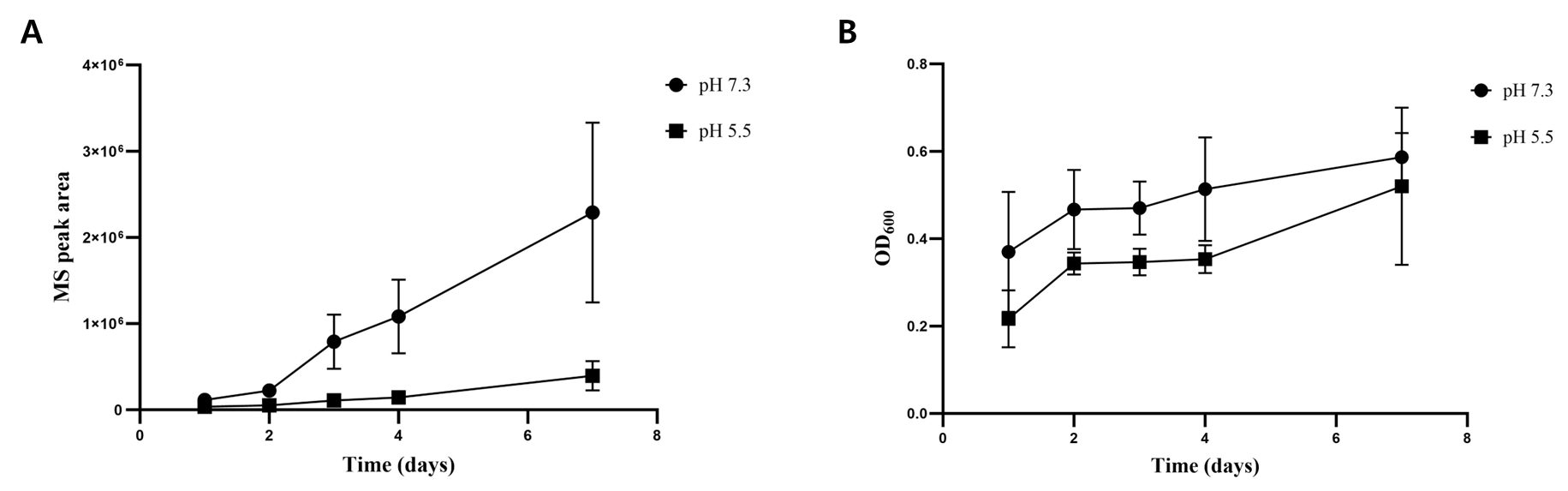


**Figure S1. MS peak areas of PAA (A) and OD_600_ values (B) over time of *C. gleum* cultures at pH 7.3 and 5.5.** Data were collected from three independent cultures at each time point and pH level.

**Figure S2. Differentially expressed genes (DEGs) of *C. gleum* after cultivation at pH 5.5 or 7.3.** Scatterplot of log_2_ fold change (FC) versus log counts per million reads (CPM) showing upregulated and downregulated genes. Positive and negative log_2_ fold-changes correspond to increased expression at pH 5.5 and 7.3, respectively. Data were obtained from single experiment.

**Figure S3.** **Survival curves of vaginal pathogens and commensal *Lactobacillus* spp. upon treatment with PAA**

**Figure S4. HRESIMS data of PAA**

**Figure S5. ^1^H NMR spectrum of PAA (500 MHz, DMSO-*d*_6_)**

**Figure S6. ^13^C NMR spectrum of PAA (125 MHz, DMSO-*d*_6_)**

**Figure S7. HMQC spectrum of PAA (500 MHz, DMSO-*d*_6_)**

**Figure S8. HMBC spectrum of PAA (500 MHz, DMSO-*d*_6_)**

**
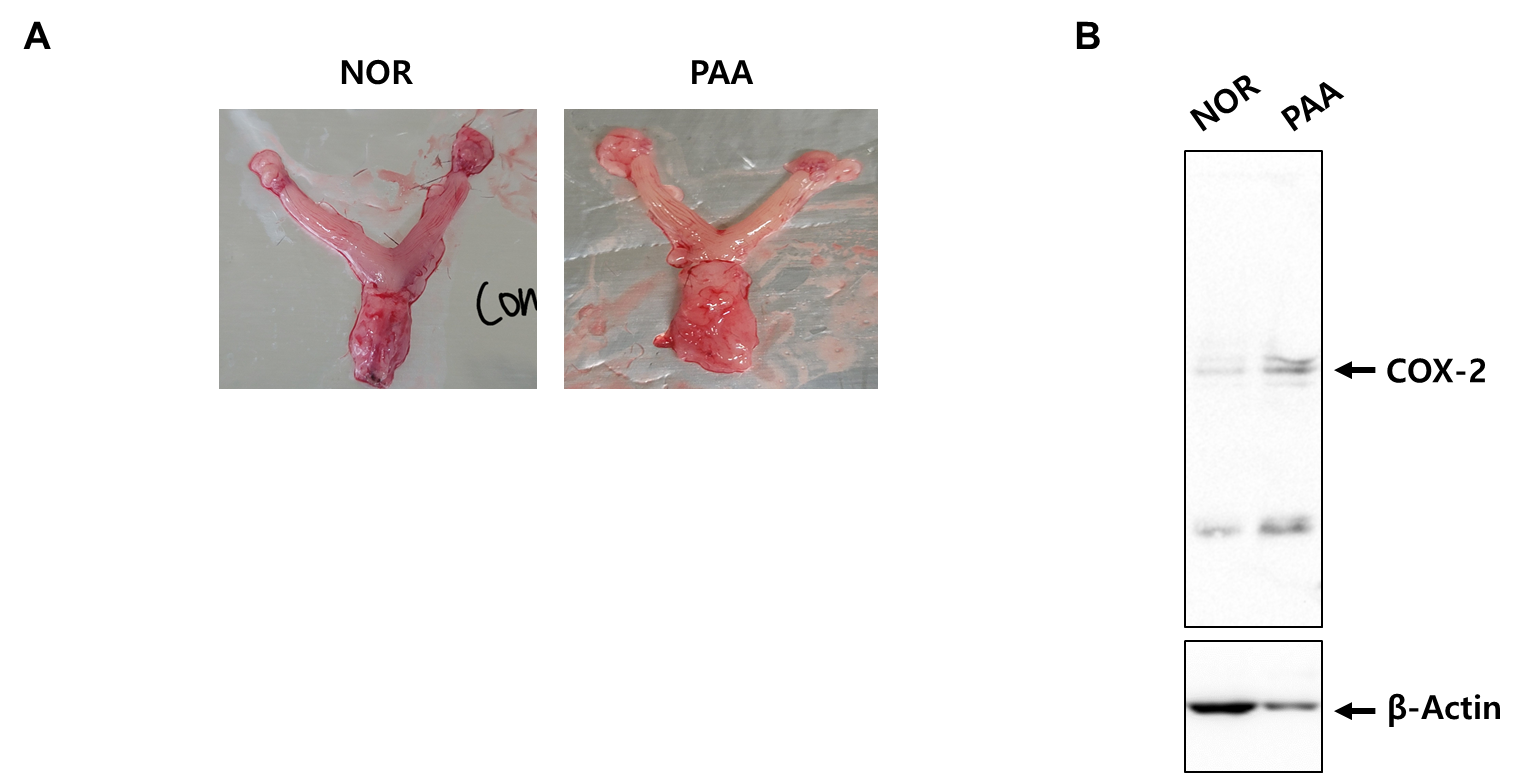
**

**Figure S9. Effects of PAA on vaginal infection and expression of inflammatory markers in female mice.** (A) Image of the vagina and uterus after treatment with PAA (1 mg/mouse). (B) Western blot analysis of the effect of PAA (1 mg/mouse) on COX-2 expression. β-Actin was used as the internal control. Data are representative of three replicate mice.
