# Supplementary material for: Phenylacetic acid, an anti-vaginitis metabolite produced by the vaginal symbiotic bacterium Chryseobacterium gleum
Source: Sci Rep. 2024 May 28;14:12226. doi: 10.1038/s41598-024-62947-7 (PMC11133378; doi:10.1038/s41598-024-62947-7)
Supplement: Supplementary file 4 — Supplementary Information 4. [file 41598_2024_62947_MOESM4_ESM.pptx]

## Slide 1
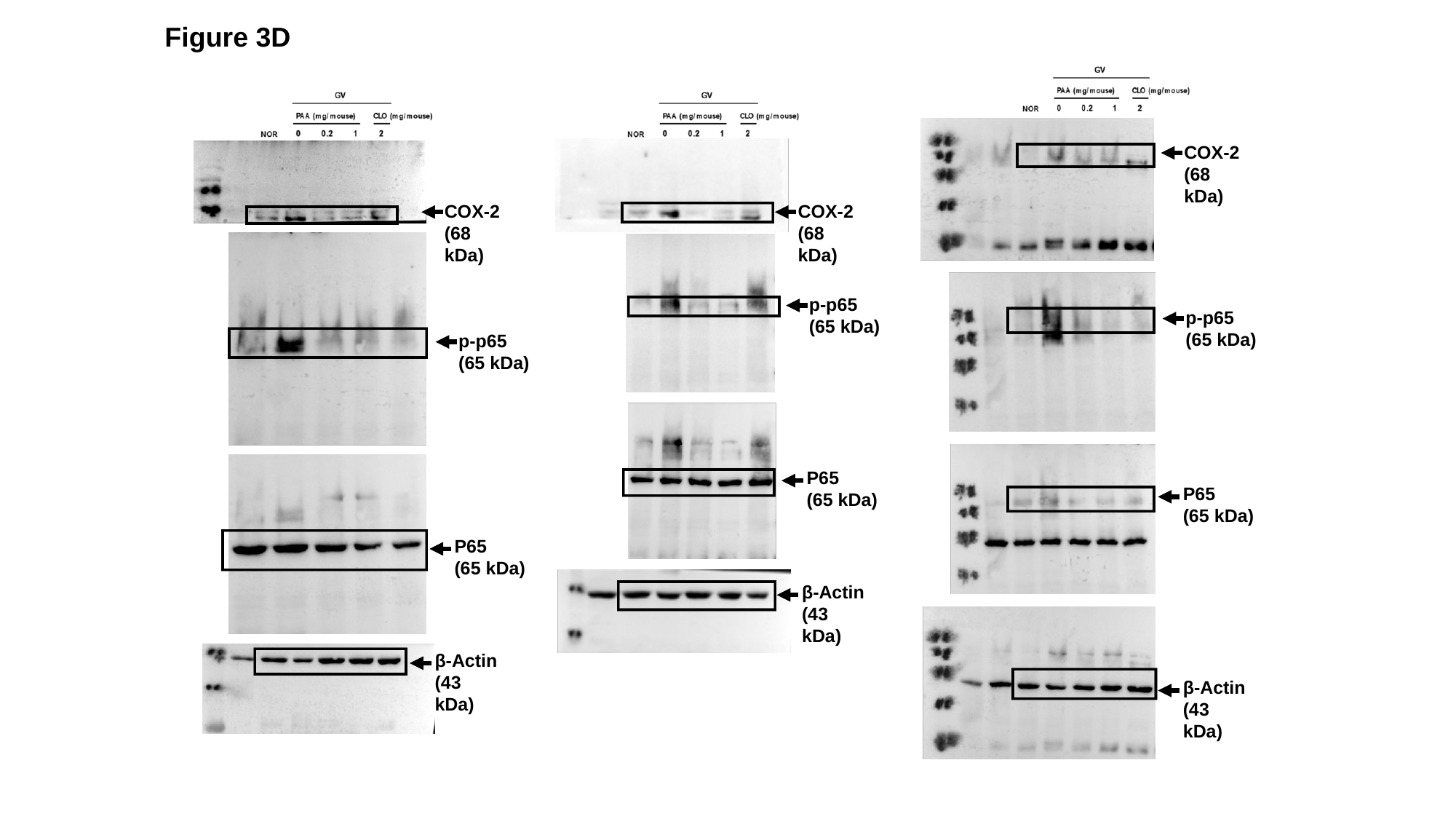

Figure 3D
COX-2
(68 kDa)
COX-2
(68 kDa)
COX-2
(68 kDa)
p-p65
(65 kDa)
p-p65
(65 kDa)
p-p65
(65 kDa)
P65
(65 kDa)
P65
(65 kDa)
P65
(65 kDa)
β-Actin
(43 kDa)
β-Actin
(43 kDa)
β-Actin
(43 kDa)

## Slide 2
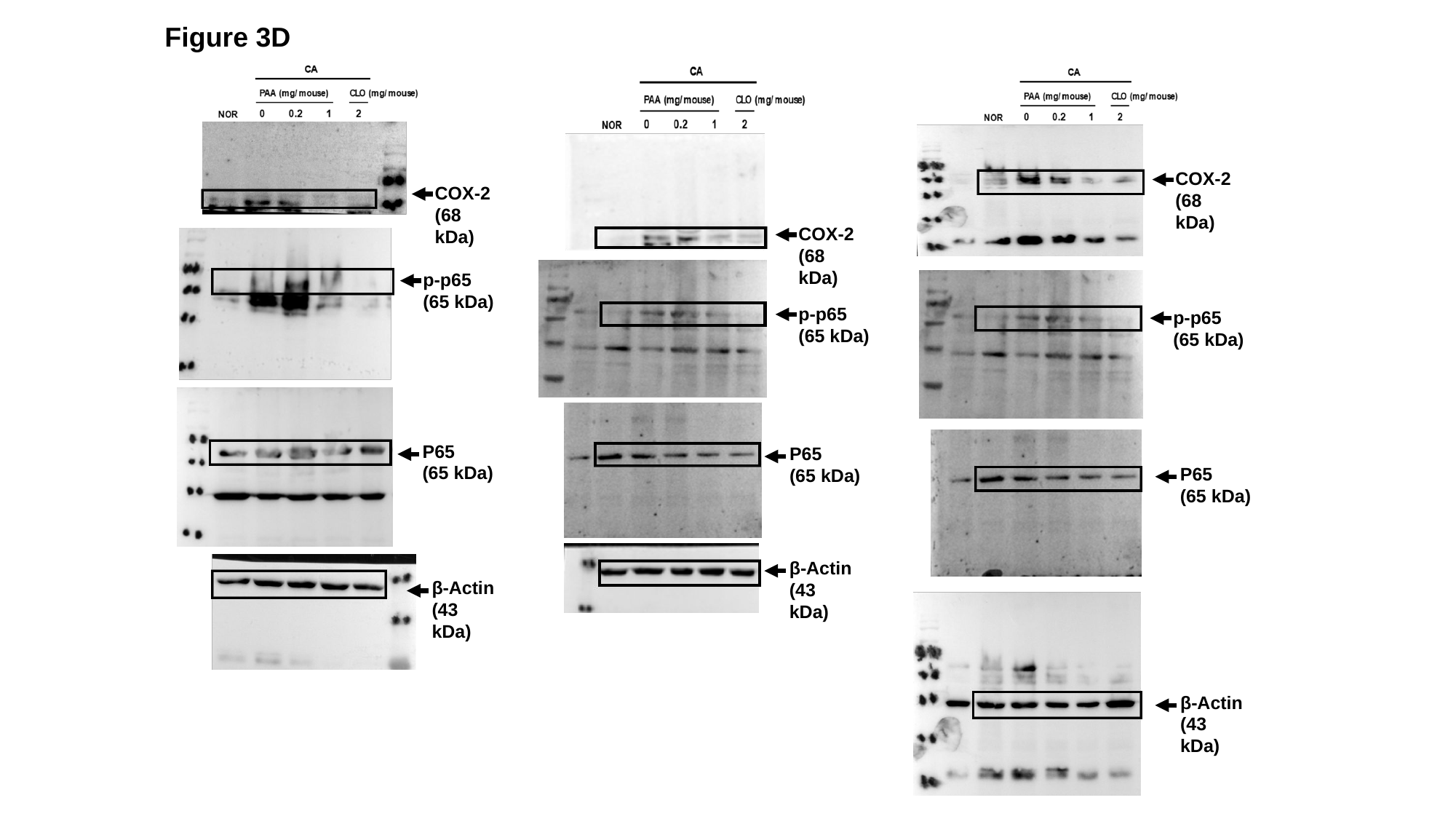

Figure 3D
COX-2
(68 kDa)
COX-2
(68 kDa)
COX-2
(68 kDa)
p-p65
(65 kDa)
p-p65
(65 kDa)
p-p65
(65 kDa)
P65
(65 kDa)
P65
(65 kDa)
P65
(65 kDa)
β-Actin
(43 kDa)
β-Actin
(43 kDa)
β-Actin
(43 kDa)

## Slide 3
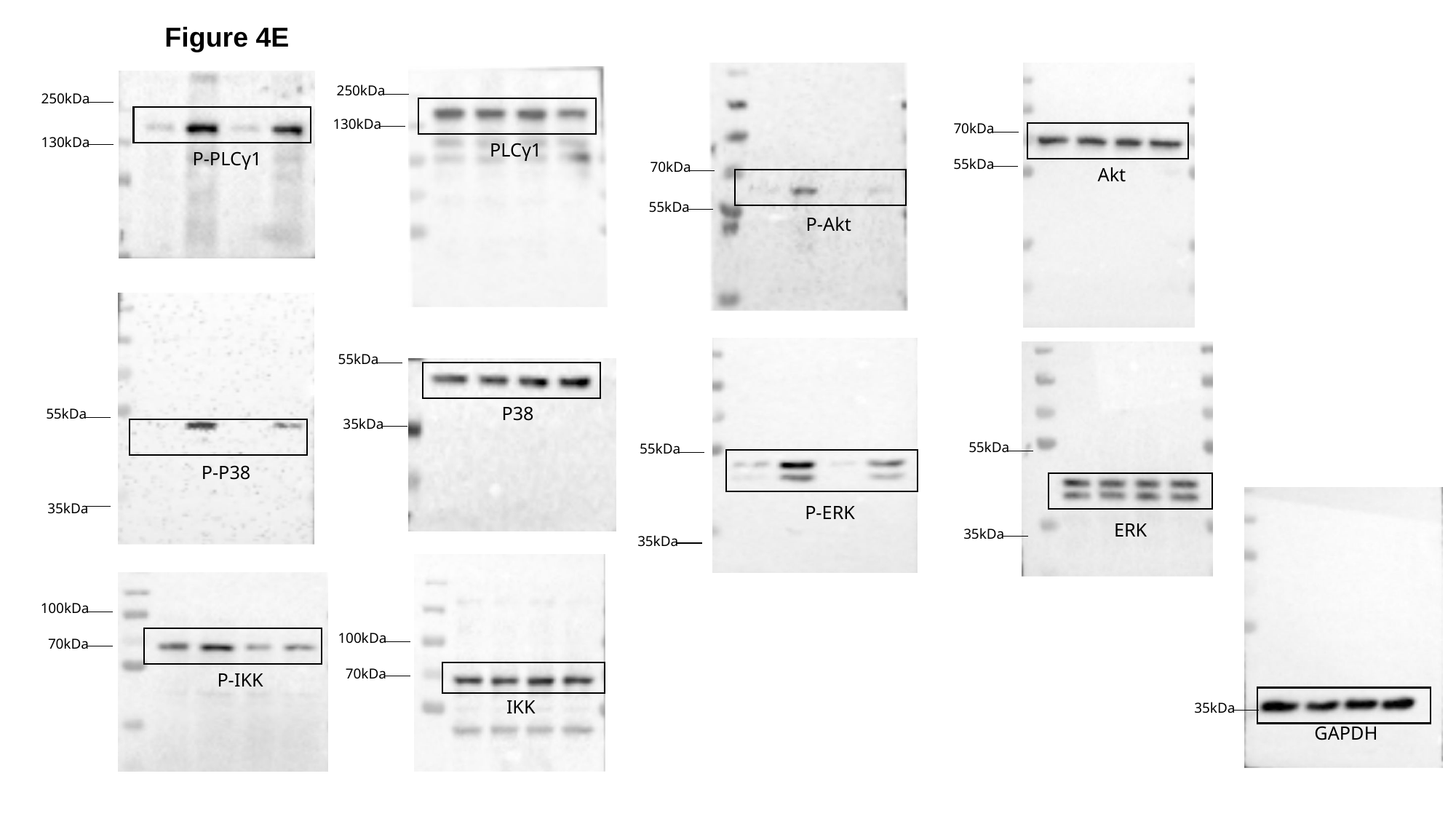

Figure 4E
70kDa
55kDa
Akt
70kDa
55kDa
P-Akt
250kDa
130kDa
PLCγ1
250kDa
130kDa
P-PLCγ1
55kDa
P-P38
35kDa
55kDa
P-ERK
35kDa
55kDa
ERK
35kDa
55kDa
P38
35kDa
35kDa
GAPDH
100kDa
70kDa
IKK
100kDa
70kDa
P-IKK
